# Commissioning and validation of a single photon beam model in RayStation for multiple matched Elekta Linacs

**DOI:** 10.1002/acm2.14485

**Published:** 2024-08-27

**Authors:** Lin Su, Ellen Huang, Devin A. Miles, Reza Farjam, Ian R. Marsh, Qiongge Li, Joseph A. Moore, Todd R. McNutt, Kai Ding, Ken Kang‐Hsin Wang, Adam Robinson, Gregory Kuri, Richard Seabrease, David P. Adam, Ryan Oglesby, Bin Shen, Binbin Wu, Junghoon Lee, Xun Jia, Sarah Han‐Oh

**Affiliations:** ^1^ Department of Radiation Oncology and Molecular Sciences Johns Hopkins University Baltimore Maryland USA; ^2^ Department of Radiation Oncology Brown University Providence Rhode Island USA; ^3^ Department of Radiation Oncology University of Minnesota Minneapolis Minnesota USA; ^4^ Department of Radiation Oncology UT Southwestern Medical Center Dallas Texas USA

**Keywords:** matched linacs, RayStation, single beam model, TPS modeling and validation

## Abstract

**Purpose:**

A single treatment planning system (TPS) model for matched linacs provides flexible clinical workflows from patient treatment to intensity‐modulated radiation therapy (IMRT) quality assurance (QA) measurement. Since general guidelines for building a single TPS model and its validation for matched linacs are not well established, we present our RayStation photon TPS modeling strategy for matched Elekta VersaHD linacs.

**Method:**

The four linacs installed from 2013 to 2020 were matched in terms of Percent Depth Dose (PDD), profile, output factor and wedge factors for 6‐MV, 10‐MV, 15‐MV, and 6‐MV‐FFF, and maintained following TG‐142 recommendations until RayStation commissioning. The RayStation single model was built to represent all four linacs within the tolerance limits recommended by MPPG‐5.a. The comprehensive validation tests were performed for one linac following MPPG‐5.a and TG‐119 guidelines, and spot checks for the other three. Our TPS modeling/validation method was evaluated by re‐analyzing the previous 103 patient‐specific IMRT/volumetric modulated arc therapy (VMAT) QA measurements with the calculated planar doses by the single model in comparison with the analysis results using four individual Pinnacle TPS models.

**Results:**

For all energies, our single model PDDs were within 1% agreement of the four‐linac commissioning measurements. The MPPG‐5.a validation tests from 5.1 through 7.5 and all TG‐119 measurements passed within the recommended tolerance limits. The IMRT QA results (mean ± standard deviation) for RayStation single model versus Pinnacle individual models were 98.9% ± 1.3% and 98.0% ± 1.4% for 6‐MV, 99.9% ± 0.1% and 99.1% ± 1.9% for 10‐MV, and 98.2% ± 1.3% and 97.9% ± 1.8% for 6‐MV‐FFF, respectively.

**Conclusion:**

We successfully built and validated a single photon beam model in RayStation for four Elekta Linacs. The proposed new validation methods were proven to be both efficient and effective.

## 1 INTRODUCTION

Modern linear accelerators (LINACs) are equipped with advanced hardware systems that can be custom‐tuned to produce high‐energy x‐ray or electron radiation beams as a user defines. This capability allows matching the beam characteristics of multiple LINACs to each other or against a pre‐configured golden beam data for treatment planning system (TPS) commissioning within the clinical tolerance limits. A single TPS model representing matched LINACs provides efficient clinical workflows, including flexible LINAC assignment for patient treatment, better balance of patient volume per LINAC, patient treatment transfer without re‐planning, re‐documentation, and physics chart check, intensity‐modulated radiation therapy (IMRT) quality assurance (QA) measurement on any matched LINAC if multi‐leaf colimmator (MLC) QA is established, patient treatment with no or minimal delay, and a back‐up solution for machine downtime.

There has been a wealth of publications on the subject of multiple‐linac beam matching and single beam model commissioning.^1^, ^2^, ^3^, ^4^, ^5^ However, the majority of these studies have focused on Varian Linacs or the Pinnacle TPS. Publications specifically addressing the commissioning of RayStation's single beam model on multiple Elekta Linacs are notably scarce. Our institution recently transitioned from the Pinnacle TPS to RayStation TPS and constructed a single model for four matched Elekta VersaHD linacs (Elekta Inc., Stockholm, Sweden). This study aims to investigate efficient and effective strategies for commissioning a single RayStation model across multiple Elekta linacs and we are eager to share our findings and experiences with the broader medical physics community.

The photon and electron single model building and validation are done separately, this paper presents the work on photon beams.

## 2 MATERIALS AND METHODS

### 2.1 Devices used

For water tank scanning, we utilized the IBA Blue phantom 2 (IBA Dosimetry, GmbH, Neu‐Isenburg, Germany). Scanning dosimeters included the IBA CC13 ion chamber, CC04 ion chamber, Photon diode detector, and electron diode detector. For validation test, CIRS Solid water phantom and cork slab were used for the point dose measurement. PTW 30013 (PTW, Freiburg, Germany) Farmer type ion chamber and IBA CC13 ion chamber was used as dosimeter for point dose measurement. The electrometer used is PTW UNIDOS E. Two‐dimensional dose distribution was measured with MapCHECK2 diode array (Sun Nuclear, Melbourne, FL, USA) with 10 cm MapPHAN built‐up. Gammex density phantom (Gammex Inc., Middleton, WI, USA) was used for CT density calibration test. Details of the detectors used in this study are listed in Table 1.

**TABLE 1:** detectors used in this study.

Detector	Vendor	Description
IBA CC13	IBA Dosimetry	0.13 cc sensitive volume ion chamber
IBA CC04	IBA Dosimetry	0.04 cc sensitive volume ion chamber
IBA EFD	IBA Dosimetry	Electron diode
IBA PFD	IBA Dosimetry	Photon diode
PTW 30013	PTW	0.6 cc sensitive volume ion chamber
MapCHECK2	Sun Nuclear	Diode array with 1527 detectors

### 2.2 Elekta VersaHD linacs match

VersaHD is a modern C‐arm medical Linac equipped with 160‐leaf Agility MLC's. Four VersaHD's in our department, commissioned between 2013 and 2020, came with five photon energies: 6 MV, 10 MV, 15 MV, 6MV‐FFFF, and 10MV‐FFF (Note: 10MV‐FFF was later decommissioned due to its limited application and suboptimal stability). During the acceptance tests, the machines were matched based on following methodology:

Percent Depth Dose (PDD) were acquired with step scan at central axis (CAX) for different energies for field size 10 × 10 cm^2^ (field size, FS = 10 × 10). Beam energy was fine tuned to match PDD 5 cm (D5), D10, D20, D10/D20 within absolute 0.5% difference for 10 × 10 cm^2^ FS. After PDD matching with FS = 10 × 10, PDD of FS = 2 × 2, FS = 4 × 4 and FS = 30 × 30 were acquired with water tank to validate the match.

Profiles were acquired with continuous scan. The beam energy and beam steering were fine‐tuned to matched flatness and symmetry. Profiles for FS = 30 × 30 for all energies at d_max_, 10 and 20 cm were matched between machines with flatness difference within 1% and symmetry < 1%. Then Profiles for FS = 10 × 10 and FS = 4 × 4 were acquired to validate matching between machines. Since the fine tuning of flatness caused subtle change of beam energy, the PDD for FS = 10 × 10 were re‐scanned for verification.

Output factors from FS = 1 × 1 to FS = 25 × 25 were measured and confirmed within 2% among machines.

Elekta uses universal wedge (wedge angle 60°). Wedge factor was tuned by tweaking the wedge position relative to the central axis. All wedge factors were tuned to within 2% among four machines.

After commissioning, all four linacs were well maintained according AAPM TG142 recommendation.^6^

### 2.3 Beam modeling in RayStation

Before beam modeling, beam data from four machines were compiled and compared. The objective was to create a model with parameters that are representative of the median values from the different machine beam parameters. Collapse Cone dose algorithm was used for the modeling.

RayStation (version 10A)^7^ provides a suite of automated modeling tools, greatly simplifying the beam modeling process. The modeling began with the Multi‐parameter auto‐modeling tool, utilizing the complete set of beam data from one machine. This tool produced a preliminary model. This model was then juxtaposed with beam data from all four machines to identify major discrepancies. Subsequently, single‐parameter auto‐modeling was applied to refine the model and reduce these discrepancies. Available auto modeling tool includes beam profile corrections, electron contamination, energy spectrum, output factor corrections, off axis softening, and so forth. After applying the single‐parameter auto‐modeling tool and achieving better alignment with the beam data, some parameters were manually adjusted to further minimize errors. The MLC and jaw position offsets, as well as the source dimensions, were iteratively evaluated for each energy by comparing the measured and computed beam profile penumbra at a depth of 10 cm.

The curve quality in RayPhysics was employed for evaluation of the agreement. For PDD data, the curve quality was assessed for both the build‐up region and the fall‐off region. For beam profiles, the curve quality was computed for the in‐field, penumbra and out‐of‐field region. The curve quality value is the Root Mean Square (RMS) difference between measured data and computed data. It can be defined by the following equation, where *N* is the total number of points included in the associated region:

$$RMS\;=\sqrt{\frac{1}{N}\sum_{i=1}^{N}{\left(\frac{Computed\;dose-measured\;dose}{maximum\;dose\;for\;measured\;curve}\right)}^{2}}\;$$

The evaluation criteria for the beam modeling of the PDD fall‐off region is 2%, and for the in‐field region of profiles, it's 3%. The model was further refined based on the results from the validation process.

### 2.4 Validation

For the Validation, the AAPM MPPG‐5a^8^ offers a comprehensive list for photon model validation. This includes basic dose algorithm validation, heterogeneity correction validation, and IMRT/VMAT dose validation. Complete full validation on all four linacs might necessitate extensive test time and significant physicist manpower. Given that all four VersaHDs are meticulously matched during commissioning and well maintained during annual QA, their performances are closely aligned. We opted to conduct full MPPG‐5a photon validation for one of the VersaHDs, and perform spot check on other machines. Moreover, all the machines were tested with simple static fields measured at their commissioning, and extensive sets of IMRT/VMAT QA cases. Finally, each linac was tested with Imaging and Radiation Oncology Core (IROC) anthropomorphic phantom as End‐to‐End test.

#### 2.4.1 MPPG‐5a validation

All MPPG‐5a validation tests are new measurements at time of this work. Four different types of validation test were performed. A brief explanation is given here, for detailed information, please refer to reference 1(MPPG‐5.a). The validation tests are summarized in Table 2.

**TABLE 2:** validation tests performed in this study.

Test type	Test indices in MPPG‐5a
TPS model comparison	5.1–5.3
General photon field test	5.4–5.8
Heterogeneity correction	6.1–6.2
IMRT/VMAT	7.1–7.5

Abbreviations: IMRT, intensity‐modulated radiation therapy; TPS, treatment planning system.

The TPS model comparison test primarily served as a data sanity check (MPPG‐5a test 5.1‐5.3). In‐house MATLAB code was employed to compare the dose in modeling model and clinical planning module. The reference calibration condition check was performed as well.

**FIGURE 1:**
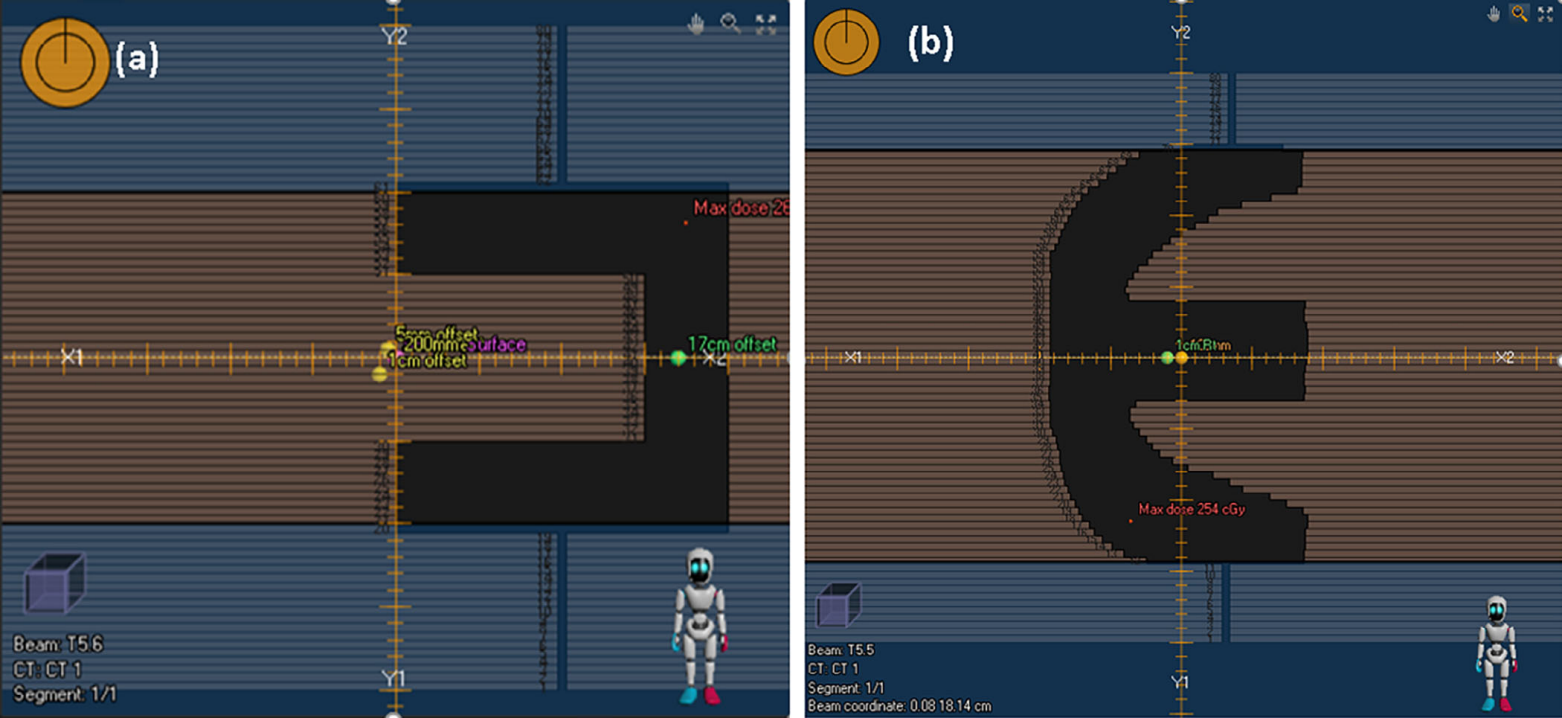
Sample validation fields in RayStation (a) Test 5.6; (b) Test 5.5.

For general photon field test, a number of field configurations different from those used for modeling were employed (MPPG‐5a test 5.4‐5.8). The dose measurements were performed with Farmer chamber at different depths and off‐axis locations. Some examples of the fields were shown in Figure 1

Heterogeneity correction validation encompassed MPPG‐5a test 6.1–6.2. test 6.1 is for CT‐density calibration validation with Gammex density phantom. Test 6.2 is the dose measurement in heterogeneous phantom made of solid water and cork.

The IMRT/VMAT validation consisted of MPPG‐5a test 7.1–7.5. Test 7.1 and 7.2 are the verifications of small field PDD and output. Test 7.3 are TG‐119 tests. For test 7.4, 10 clinical IMRT cases of different treatment sites were calculated and measured with MapCHECK2. Test 7.5 is the external reviewed End‐to‐End test, this test was performed for each linac and will be described in Section 2.4.4.

#### 2.4.2 Simple fields validation

In additional to the MPPG‐defined validation fields, an assortment of fields used for validation during each machine's commissioning were calculated in RayStation and compared with measurement for further validation. For each FF energy, there are 12 non‐wedge fields and 10 wedge fields, ion chamber measurements were at 5 cm depth, 15 cm depth at central axis, and 10 cm depth 5 cm off axis.

#### 2.4.3 More comprehensive IMRT/VMAT QA validation

The vast majority of our treatments are IMRT/VMAT. To bolster confidence in the IMRT/VMAT modeling, 103 IMRT or stereotactic body radiotherapy (SBRT) patients were assessed. These patients were randomly selected from all individuals treated 3 months before the study (January 2023), encompassing different energies, treatment sites, and modality (IMRT or SBRT). The selected cases were planned in pinnacle v16.2 and QAed with MapCHECK2. For this test, selected RTPlan DICOM files were exported from pinnacle and imported to RayStation and recalculated in the new single VersaHD model with Collapse Cone algorithm. The calculated plan dose was compared with initial MapCHECK2 measurement. There were 58 6‐MV cases, 8 10‐MV cases, and 37 6‐MV‐FFF cases. After the clinical application of RayStation, we collected the QA results from June 2023 to August 2023, including 228 6‐MV cases, 120 6‐MV‐FFF cases, and 45 10‐MV cases. For 6 MV and 10 MV cases, 3%/3 mm, 10% threshold, global normalized were used; for 6‐MV‐FFF cases, 2%/2 mm, 10% threshold, global normalized were used.

#### 2.4.4 Externally reviewed end‐to‐end test

Upon successful completion of prior validations, four IROC anthropomorphic phantoms (Head and Neck, Lung, Spine, Prostate) were requisitioned for four linacs to execute end‐to‐end test. Each phantom contained embedded films and thermoluminescent dosimeters (TLD) dosimeters for IROC analysis. The plans were simed by therapists, planned by dosimetrists, checked by physicists, and delivered by therapists, and results were sent to IROC for independent evaluation. For the sake of consistency and to emulate real‐life scenarios, each phantom was paired with a linac that matched its specific treatment focus. The selection of beam energy for the treatment was in alignment with the conventional clinical practice: 6‐MV for the Head and Neck region, 6‐MV FFF for Spine and Lung SBRT treatments, and 10‐MV for Prostate.

## 3 RESULTS

### 3.1 VersaHD linacs match

The comparison of the four machines revealed close matches. Key results are: Average variance in PDD D10, D20, D10/D20 across the four machines: 0.3% (max: 0.9%). All profile flatness and symmetry differences remained within 1%. Output factors for FS ≥ 3 × 3 and the wedge factor were both within a 1% range.

### 3.2 Beam modeling in RayStation

The final modeled data exhibited a good match with the measurement data. For the falloff region in photon PDD curves: max differences (RMS) for 6 MV, 10 MV, 15 MV, and 6 MV‐FFF stood at 0.4%, 0.3%, 1.0%, and 0.4%, respectively. For wedge fields of 6 MV, 10 MV, and 15 MV, the max differences were 0.9%, 0.9%, and 1.4% respectively. The in‐field region of photon profile curve for different field size were summarized in Table 3.

**TABLE 3:** in‐field region of photon profile curve difference of model and measurement for all field sizes (mean ± standard deviation).

6 MV	10 MV	15 MV	6 MV‐FFF	6 MV wedge	10 MV wedge	15 MV wedge
0.7% ± 1.0%	1.0% ± 0.4%	0.9% ± 0.3%	0.5% ± 0.4%	1.3% ± 0.5%	2.0% ± 0.8%	1.3% ± 0.7%

### 3.3 Model validation

#### 3.3.1 MPPG‐5a validation

In Test 5.1, our in‐house MATLAB code demonstrated that the dose distributions between the planning and physics modules were consistent. Test 5.2 revealed a minimal calibration deviation, with the maximum deviation from 1 cGy/MU being 0.2%. Test 5.3 showed a commendable passing rate of 98.3% for a 2%/2 mm comparison of PDD, profile, and beam model. The deviation in output factors was comfortably within the 2% tolerance.

In Tests 5.4 to 5.8, non‐wedge fields displayed an average difference of 0.8% and a maximum of 1.8%. In contrast, wedge fields showed a slightly higher average difference of 1.8%, but it remained under the MPPG‐5a tolerance threshold of 5%.

Test 6.1 indicated an average difference of 1.0% between RS density and real density, with the maximum deviation being 1.9%. Test 6.2, conducted across four energy levels at varying depths, yielded an average difference of 1.0% and a maximum of 2.9%, well within the MPPG‐5a tolerance of 3%.

In Tests 7.1 and 7.2, the 2%/2 mm comparison for a 2 × 2 cm^2^ field yielded passing rates ranging between 95.9% and 99% across four energies. Test 7.3, focusing on TG119 defined plans, showcased a mean passing rate of 98.7% for the 2%/2 mm criterion and 99.4% for the 3%/3 mm criterion. Test 7.4 solidified these findings with a mean passing rate of 99.4% for the 3%/3 mm criterion.

#### 3.3.2 Simple field validation

For non‐wedge fields, the average difference between ion chamber measurements and RayStation calculations was a mere 0.7%, with a maximum deviation of 2.7%. Wedge fields showed a similar trend, with an average difference of 1.0% and a peak at 3.5%. It's worth noting that the most significant deviations occurred at deeper depths and off‐axis positions, still within the MPPG‐5a recommended 5% tolerance.

#### 3.3.3 More comprehensive IMRT/VMAT QA validation

Table 4 offers a comparison of IMRT/VMAT results comparison for RayStation and Pinnacle TPS across various energies. This first column is for previously treated pinnacle plans calculated in RayStation; the second column is previously treated pinnacle plans; the third column is the new clinical RayStation plans The gamma analysis results for 6 MV and 10 MV utilized a 3%/3 mm criterion, while the 6 MV‐FFF used a 2%/2 mm criterion. The results consistently remained within the standard deviation, affirming the consistency and reliability of both systems. Meanwhile, the RayStation plans has slightly higher QA pass rate than Pinnacle plans.

**TABLE 4:** Comparison of IMRT QA passing rates for RaySation versus Pinnacle (mean ± SD).

Energy	Pinnacle plans calculated in RayStation	Pinnacle plans	New RayStation plans
6 MV	98.9% ± 1.3%	98.0% ± 1.4%	99.4% ± 0.9%
10 MV	99.9% ± 0.1%	99.1% ± 1.9%	99.7% ± 0.7%
6 MV FFF	98.2% ± 1.3%	97.9% ± 1.8%	98.6% ± 1.3%

*Note*: The Second column is for previously treated pinnacle plans calculated in RayStation; the third column is for previously treated pinnacle plans; the fourth column is for RayStation IMRT plans.

Abbreviations: IMRT, intensity‐modulated radiation therapy; QA, quality assurance.

#### 3.3.4 Externally reviewed end‐to‐end test

Table 5 showcases the IROC phantom results, reflecting the precision of the VersaHD linacs across diverse phantom types and energy beams. The results from the TLD reading ratios and film gamma index analysis further emphasized the linac's accuracy and the reliability of the integrated RayStation model.

**TABLE 5:** Results for end‐to‐end test with IROC phantom.

			TLD reading ratio IROC/Inst		Film gamma index analysis			
Linac ID	Phantom type	Beam Energy	Min	M6ax	criteria	Axial	Coronal	Sagittal
1	Head & Neck	6 MV	0.96	1.00	7%/4 mm	99%	N/A	92%
2	Lung	6 MV‐FFF	0.99	1.04	7%/5 mm	100%	100%	99%
3	Spine	6 MV‐FFF	0.97	1.00	5%/3 mm	91%	N/A	98%
4	Prostate	10 MV	1.00	1.01	7%/4 mm	N/A	100%	100%

## 4 DISCUSSION

Unlike Varian, which provide “golden beam data” to streamline linac matching and commissioning, Elekta used to lack a comparable feature until the recent introduction of the Accelerated‐go‐live (AGL) System. Regrettably, all four of our Elekat VersaHD Linacs were commissioned before AGL is available, necessitating our physicists to exert extra effort in matching these machines. While these linacs are closely matched, we constructed individual beam models for each machine for optimal representation during commissioning. However, over years of clinical usage, these individual models presented logistical challenges in patient transfer and IMRT QA, particularly during the COVID‐19 pandemic. As our department transitioned from Pinnacle to RayStation, we conceived the idea of creating a single model for all Elekta Linacs. This single model approach would enhance our clinic's efficiency, enabling seamless machine‐switching for treatments and facilitating IMRT QA on any machine.

The primary goal of this work is to explore a practical method for constructing a single RayStation model that can represent multiple Elekta linacs with minimal new scan data. Our well‐maintained linacs, combined with RayStation's robust auto‐modeling features, expedited the modeling process. We first undertook a comprehensive MPPG‐5a validation on one machine, which spanned 2 days and yielded satisfactory results. Conducting identical tests on the remaining three machines would have extended the process by another week. Considering our machines' close matching, we didn't anticipate significant variances in the validation outcomes, especially as most MPPG‐5a validations involve relatively simple static open‐field measurements. Given that a majority of our treatments are IMRT/VMAT and recognizing the potential challenges in modeling accuracy within the penumbra area for high modulation IMRT, we opted to bypass the full MPPG‐5a validation for the three other machines. Instead, we emphasized an exhaustive IMRT/VMAT validation with over 100 clinical cases. This approach was both time‐efficient and offered a more stringent validation. We advocate this approach for other institutions who want to build single TPS model for closely‐matched linacs.

The novelty of our work is outlined as follows: A typical way of setting up a single TPS model is to adjust a newly commissioned machine to match the single model. Our approach commissioned a single model after the four matched Eleketa linacs were already commissioned as four individual models in a different TPS, in our case Pinnacle, and were already treating patients daily. These two constraints often happen in the clinic when the TPS is switched from one vendor to another. Our work demonstrated an efficient process of building a single RayStation model using the minimally acquired new beam dataset from one machine based on the RayStation requirement and the four beam datasets acquired for Pinnacle commission. A draft RayStation model was built on the new dataset and iteratively adjusted against the four Pinnacle beam datasets such that the model can represent all four linacs within the tolerance limits recommended by MMPG5a without adjusting the beam parameters. In addition, our study proves that an individual TPS model is not clinically superior to a single model by comparing the IMRT QA passing rates calculated by the two models.

Pinnacle TPS has been employed in our institute for more than two decades. While Pinnacle remains a competent TPS with sophisticated physics modeling functionalities, it is hindered by an outdated user interface and falls short in offering some automated features. RayStation, an emerging newcomer in the TPS arena, boasts automation and a user‐centric interface. We particularly value the auto‐modeling tools in RayPhysics, which curtail modeling time and amplify accuracy. Additionally, RayStation's GPU server‐driven dose computation speed outpaces Pinnacle by 1−2 orders of magnitude.

Nevertheless, RayStation is not without areas for improvement. For instance, it lacks kernel tilting in the dose engine, which results in more pronounced errors in the profiles for larger fields and greater depths, particularly in the penumbra area. Fortunately, such conditions are infrequent in IMRT treatment. Another example is the wedge model in RayStation, which is tethered to the open field model of the corresponding energy, leaving us with limited room for fine‐tuning, that's why our wedge model agreement is generally lower than open field model (but still within tolerance of MPPG‐5a). In contrast, Pinnacle's wedge model operates independently from its open field model, granting physicists greater flexibility in modeling. Adopting a similar independent approach in RayStation would likely improve the model fidelity of its wedge fields. Lastly, we have observed discrepancies in the dose calculations near high *Z* materials, stemming from RayStation's photon beam's minimum energy bin being set at 0.5 MeV, in comparison to Pinnacle's 0.25 MeV. This became particularly evident in our IMRT QA results when we analyzed doses computed in MapCHECK2, which has a central diode array. Pinnacle's calculations presented a “ripple‐style” dose profile due to the influence of high *Z* materials on low energy photons, whereas RayStation's dose profiles, given its 0.5 MeV minimum energy, appeared artificially smoother.

## 5 CONCLUSION

We successfully commissioned and validated RayStation single photon model on four of our Elekta VersaHD linacs. The model performance was comparable or better than our previous individual models. We proposed, tested, and affirmed a novel method for TPS validation for multiple matched linacs. We are confident that the insights and methodologies outlined in our experience will serve as a useful guide for medical physicists in other institutions.

## AUTHOR CONTRIBUTIONS

All authors contributed in the study design, data collection and analysis, manuscript preparation, and its final review.

## CONFLICT OF INTEREST STATEMENT

The authors declare no conflict of interest.

## Data Availability

The data that support the findings of this study are available on request from the corresponding author. The data are not publicly available due to privacy or ethical restrictions.

## References

[1] Sjöström D , Bjelkengren U , Ottosson W , Behrens CF . A beam‐matching concept for medical linear accelerators. Acta Oncol. 2009;48(2):192‐200. 18752079 10.1080/02841860802258794

[2] Beyer GP . Commissioning measurements for photon beam data on three TrueBeam linear accelerators, and comparison with Trilogy and Clinac 2100 linear accelerators. J Appl Clin Med Phys. 2013;14(1):273‐288. 10.1120/jacmp.v14i1.4077 PMC5714054 23318395

[3] Sarkar B , Manikandan A , Nandy M , et al. A mathematical approach to beam matching. Br J Radiol. 2013;86(1031):20130238. 23995874 10.1259/bjr.20130238 PMC3830429

[4] Xu Z , Warrell G , Lee S , et al. Assessment of beam‐matched linacs quality/accuracy for interchanging SBRT or SRT patient using VMAT without replanning. J Appl Clin Med Phys. 2019;20(1):68‐75. 10.1002/acm2.12492 PMC6333115 30402983

[5] Frigo SP , Ohrt J , Suh Y , Balter P . Interinstitutional beam model portability study in a mixed vendor environment. J Appl Clin Med Phys. 2021;22(12):37‐50. 10.1002/acm2.13445 PMC8664150 34643323

[6] Klein EE , Hanley J , Bayouth J , et al. Task Group 142 report: quality assurance of medical accelerators. Med Phys. 2009;36(9):4197‐4212. 19810494 10.1118/1.3190392

[7] Bodensteiner D . RayStation: external beam treatment planning system. Med Dosim. 2018;43(2):168‐176. 29650302 10.1016/j.meddos.2018.02.013

[8] Smilowitz JB , Das IJ , Feygelman V , et al. AAPM Medical Physics Practice Guideline 5.a.: commissioning and QA of Treatment Planning Dose Calculations — Megavoltage Photon and Electron Beams. J Appl Clin Med Phys. 2015;16(5):14‐34. 26699330 10.1120/jacmp.v16i5.5768 PMC5690154

